# Determination of elastic moduli of elastic–plastic microspherical materials using nanoindentation simulation without mechanical polishing

**DOI:** 10.3762/bjnano.12.17

**Published:** 2021-02-19

**Authors:** Hongzhou Li, Jialian Chen

**Affiliations:** 1 College of Environmental Science and Engineering, Engineering Research Center of Polymer Resources Green Recycling of Ministry of Education, Fujian Normal University, Fuzhou 350007, China

**Keywords:** elastic–plastic, microsphere, nanoindentation, Oliver–Pharr method, simulation

## Abstract

When using the Oliver–Pharr method, the indented specimen is assumed to be a perfectly flat surface, thus ignoring the influences of surface roughness that might be encountered in experiment. For nanoindentation measurements, a flat surface is fabricated from curved specimens by mechanical polishing. However, the position of the polished curved surface cannot be controlled. There are no reliable theoretical or experimental methods to evaluate the mechanical behavior during nanoindentation of an elastic–plastic microsphere. Therefore, it is necessary to conduct reliable numerical simulations to evaluate this behavior. This article reports a systematic computational study regarding the instrumented nanoindentation of elastic–plastic microspherical materials. The ratio between elastic modulus of the microsphere and the initial yield stress of the microsphere was systematically varied from 10 to 1000 to cover the mechanical properties of most materials encountered in engineering. The simulated results indicate that contact height is unsuitable to replace contact depth for obtaining the indentation elastic modulus of microspherical materials. The extracted elastic modulus of a microsphere using the Oliver–Pharr method with the simulated unloading curve depends on the indentation depth. It demonstrates that nanoindentation on microspherical materials exhibits a “size effect”.

## Introduction

Instrumented nanoindentation is the most commonly used technique for the characterization of the mechanical behavior of filaments [1], thin films [2], microplastics, coatings, powders, small crystals, and other materials at small scales. One of the great advantages of the technique is that many mechanical properties of materials can be determined from the analysis of indentation load–displacement data alone. This avoids the need to measure the area of indentation by imaging and facilitates the measurement of properties at the sub-micrometer scale. During nanoindentation, a diamond indenter with a geometry known to high precision is pressed into the surface of the specimen with increasing force or displacement. After force or displacement reached a specified value, the load is withdrawn. During the loading–unloading process, force–displacement curves are recorded. Taking one complete cycle of loading and unloading data, three quantities are measured. One is the maximum load, another is the maximum displacement *h*_max_ (the maximum displacement of the indenter relative to the initial undeformed surface), and the third is the unloading stiffness. The initial unloading stiffness is used to extract the elastic modulus of the specimen via the well-known Oliver–Pharr method [3–4].

Cheng and Cheng derived several scaling relationships for conical indentation in elastic–plastic solid materials with work hardening via dimensional analysis [5]. Oliver and Pharr reviewed the methodology of measuring elastic modulus and hardness by instrumented indentation [6]. The elastic and plastic properties of materials when employing a sharp indenter (geometrically self-similar indenters such as Vickers, Pyramids, Berkovich, or Cones) may be computed from a single load–displacement curve through a general theoretical framework proposed by Giannakopoulos and Suresh [7]. Their procedure is usable to precisely calculate the indentation response from a given set of elastic–plastic properties (forward algorithms), and to extract elastic–plastic properties from a given set of indentation data (reverse algorithms) [8]. Pileup (or sink-in) results in contact areas bigger (or smaller) than the cross-sectional area of the indenter at a specified depth. These effects can result in measurement errors of mechanical properties [9]. Without taking into account that the real contact area and the cross-sectional area of the indenter are different, the measured indentation modulus/hardness would be too high in the case of pileup and too low in the case of sink-in [10]. Nix and Gao deduced the theory of strain gradient plasticity to interpret the “size effect” of indentation as an increase in physical quantity with the decreasing depth of penetration [11]. Experimental results show that the size effect of indentation for pyramidal and spherical indenters can be correlated [12]. For a spherical (parabolic) indenter, hardness does not depend on depth, but on the radius of the indenter. Therefore, for spherical indentation, the radius of the impression rather than the depth of penetration determines the size effect of indentation [12]. Swadener et al. pointed out that, for some cases, the hardness is decreased with decreasing depth due to the predicted decrease in dislocation density [12].

The indented specimen is assumed to be a perfectly flat surface for the Oliver–Pharr method, thus ignoring the influences of surface roughness that might be encountered in experiment. For nanoindentation measurements, a flat surface is fabricated from curved specimens by mechanical polishing. However, the position of the polished curved surface cannot be controlled [13]. Small-scale microplastics with curved structures require material characterization. The materials properties are not affected by the geometry of the specimen, but the Oliver–Pharr procedure to obtain material properties will vary depending on the geometry of the specimen. There has been no reliable theoretical and experimental method to evaluate the mechanical behavior during nanoindentation of a curved specimen. Therefore, it is necessary to conduct reliable numerical simulations to evaluate the mechanical behavior of nanoindentation on an elastic–plastic microspherical material. The numerical simulations are usually carried out via the finite element method [14–25]. Using finite element simulation, Li et al. found that both loading curve and unloading curve at any depth can be generated from one indentation depth by scaling *P* ∝ *h*^2^ for the indentation tests of PMMA thin films with a Berkovich indenter [1–2]. The loading curve can be described by the formula [1–2]:

[1] $$P=\alpha \frac{E\tan\phi }{\left(1-\nu^{2}\right)}h^{2},$$

where *P* is the indenter load, α is a material constant, *E* and ν are the elastic modulus and Poisson’s ratio of the specimen, ϕ is the half-apical angle of the indenter, and *h* is the displacement of the indenter relative to the initial undeformed surface. It is not easy to find an explanation why different unloading curves have this relationship of *P* ∝ *h*^2^.

In this study, the finite element method has been used to systematically investigate the mechanical behavior of nanoindentation on elastic–plastic microspherical materials. The elastic modulus of nanoindentation was calculated via the Oliver–Pharr method. We found that the contact height [26] is unsuitable to replace the contact depth for obtaining the indentation elastic modulus of microspherical materials.

## Theoretical Method

The analysis of Sneddon for the indentation of an elastic half space by a flat, cylindrical punch leads to a simple relation between *P* and *h* of the form [27]

[2] $$P=\frac{4Ga}{1-\nu }h,$$

where *a* is the radius of the cylinder and *G* is the shear modulus. Noting that the contact area (i.e., the projected area or cross-sectional area of elastic contact) *A* is equal to π*a*^2^ and that the shear modulus is equal to *E*/[2(1 + ν)], differentiating *P* with respect to *h* leads to

[3] $$S=\frac{\text{d}P}{\text{d}h}=\frac{2E\sqrt{A}}{(1-\nu^{2})\sqrt{\pi }},$$

where *S* = d*P*/d*h* is the initial stiffness of the unloading curve, defined as the slope of the upper portion of the unloading curve during the initial stages of unloading (also called contact stiffness), and *E* is the elastic modulus of specimen. For a Berkovich indenter, the half-apical angle is 70.3°, and the area-to-depth relationship, also known as the area function, is given by

[4] $$A=24.5h_{\text{c}}^{2},$$

where *A* is the cross-sectional area of the indenter at contact depth *h*_C_, a distance that is measured vertically from its tip. Knowing the contact depth and the shape of the indenter, determined through the “area function”, the contact area is then determined. If contact stiffness and contact area are known, Equation 3 and Equation 4 can be used to determine the elastic modulus of a material.

Effects of non-rigid indenters on the load–displacement behavior can be effectively accounted for by defining an effective elastic modulus through Equation 5:

[5] $$\frac{1}{E_{\text{eff}}}=\frac{\left(1-\nu^{2}\right)}{E}+\frac{\left(1-\nu_{i}^{2}\right)}{E_{i}},$$

where *E_i_* and ν*_i_* are the elastic modulus and Poisson’s ratio of the indenter. If the indenter is a rigid body (i.e., *E_i_* = ∞), for any axisymmetric indenter, the effective elastic modulus *E*_eff_ can be derived as [6]

[6] $$E_{\text{eff}}=\frac{\sqrt{\pi }}{2}\frac{S}{\sqrt{A}}.$$

Combining Equation 5 and Equation 6, one obtains

[7] $$E=E_{\text{eff}}(1-\nu^{2})=\frac{\sqrt{\pi }}{2}\frac{S}{\sqrt{A}}(1-\nu^{2}).$$

The elastic modulus extracted from the Oliver–Pharr method depends on the initial stiffness of the unloading curve and the projected area of the indentation at the contact depth *h*_C_. To correct the Oliver–Pharr solution accounting for the radial displacements, Hay et al. used the finite element method to calibrate Equation 7 and included a “correction factor” β [28]. The correction factor depends on the half-apical angle of the indenter and the Poisson’s ratio of the material

[8] $$S=\beta \frac{2E\sqrt{A}}{(1-\nu^{2})\sqrt{\pi }},$$

where β is the correction factor, and *E* is the elastic modulus extracted according to Equation 8. Oliver and Pharr proposed that β = 1.05 with a potential error of approximately ±0.05, based on their analysis of available results 1.0226 ≤ β ≤ 1.085 from experiments and finite element calculations [6].

## Finite Element Method

**Figure 1:**
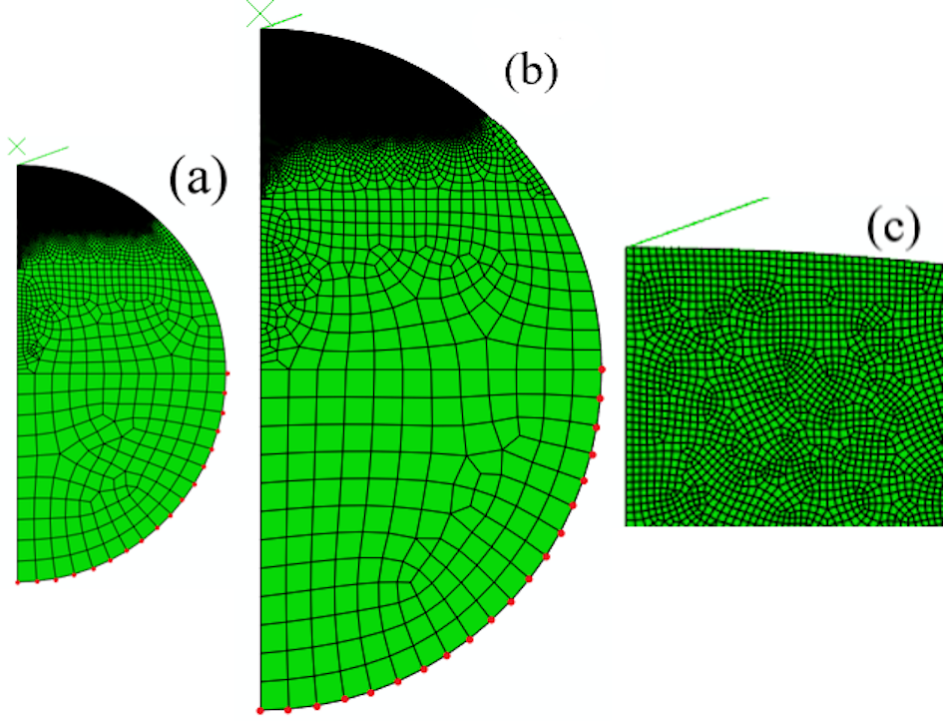
Finite element mesh: (a) one half of a microsphere with 11.5 µm radius; (b) one half of a microsphere with 23 µm radius; (c) enlargement of the refined mesh at the vicinity of conical indenter.

To evaluate the mechanical behavior of microspherical materials and to reduce the number of experimental tests, finite element simulations are used to calculate the load–displacement curves of nanoindentation during loading and unloading. The unloading curve is used to determine the elastic modulus of a material via the well-known Oliver–Pharr method. Finite element analyses of nanoindentation tests were carried out on isotropically linear elastic and isotropically perfectly plastic microspherical materials. Based on symmetry, only one half of the microsphere was modeled. Figure 1a and Figure 1b show the two 2D axisymmetric finite element models of a microsphere with 11.5 µm radius and a microsphere with 23 µm radius, respectively, in which two-dimensional CAX4R (continuum, axisymmetric, quadrilateral four-node reduced integration) and CAX3 elements were used in the mesh discretization of the microspherical materials. The whole model in Figure 1a consists of 28,651 elements and 28,538 nodes. In order to reduce the calculation time as well as to simulate the nanoindentation behavior more accurately, a finer mesh with a higher density of elements close to the contact region, as shown in Figure 1c, and a gradually coarser mesh further away from the contact region were used. The nodes in red shown in Figure 1a and Figure 1b are fixed in all directions. The mesh was well tested for convergence, that is, further refinement would not result in improving the accuracy of the simulated results. A rigid conical indenter with a half-apical angle of 70.3° was set on the top of the microsphere. It was assumed that a Berkovich indenter can be adequately modeled by an axisymmetric conical indenter since its depth-to-area relation is the same as that of an actual Berkovich indenter. The indentation was displacement-controlled by imposing a vertical displacement on the rigid conical indenter. The contact between the indenter and the microsphere was frictionless.

**Figure 2:**
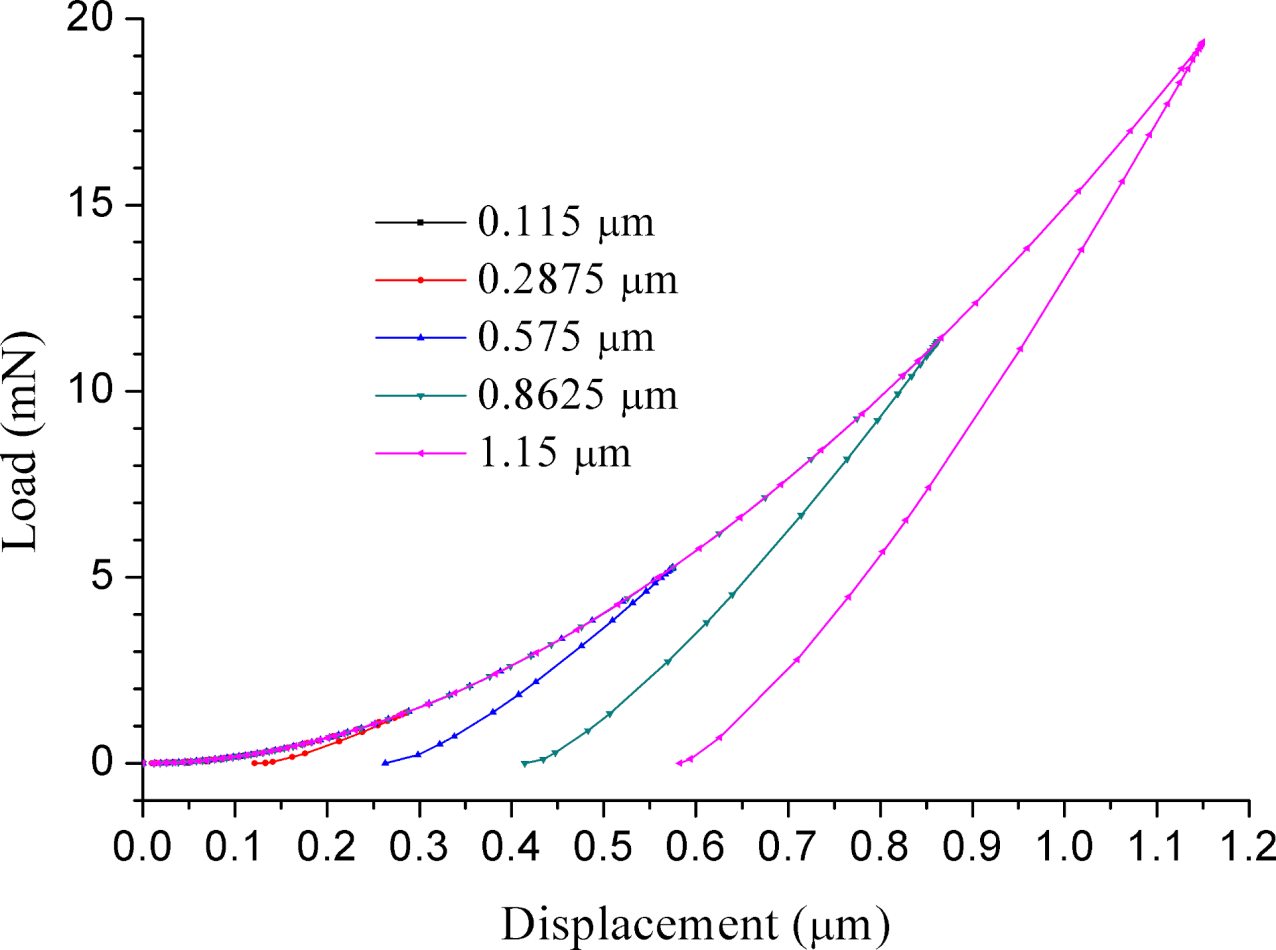
Loading and unloading curves for different indentation depths for *E*/σ_y_ = 10.

The calculation steps of nanoindentation simulation can be described as follows [1–2]:

1. Construct an axisymmetric model of a microsphere and generate its finite element mesh.
2. Assemble a finite element model by placing a rigid Berkovich indenter at the top of the microsphere as shown in Figure 1.
3. Impose a specified vertical displacement on the indenter using incremental steps.
4. After attaining the maximum displacement, the vertical displacement of the indenter is gradually reduced and is unloaded to zero using another incremental step.
5. Calculate the force acting on the indenter to obtain the loading curve and unloading curve as shown below in Figure 2.

## Results and Discussion

Dimensional analysis is widely used as a guideline for evaluating indentation tests and is also used here. Yan established a set of non-dimensional relations for conical indentation on a homogeneous, isotropic semi-infinite flat substrate, including the quantity *E*/σ_y_ [15]. σ_y_ is the initial yield stress of a linear elastic, perfectly plastic material. σ_y_/*E* is the initial yield strain. Phadikar showed that *h*_max_/*R* (*R* is the radius of a microsphere) is an appropriate non-dimensional factor [18]. Therefore, we selected the quantities *E*/σ_y_ and *h*_max_/*R* to present our results. In order to examine the effect of *E*/σ_y_ on indentation, the Poisson’s ratio of the microsphere was set to be 0.2. The ratio between elastic modulus and yield stress of a microsphere, *E*/σ_y_, was systematically varied between 10 and 1000 to cover the mechanical properties of the materials most commonly encountered in engineering. For example, an elastic–plastic material with *E*/σ_y_ = 70 and *E*/σ_y_ = 300 is fairly typical for a polymer and aluminum, respectively (low-density polyethylene with *E* = 1.37 GPa, σ_y_ = 20 MPa, and *E*/σ_y_ ≈ 69; aluminum with *E* = 70 GPa, σ_y_ = 228 MPa, and *E*/σ_y_ ≈ 307).

Figure 2 shows curves of the microsphere with 11.5 µm radius being indented to different depths by a rigid conical indenter to produce different maximum loads. The loading curves for different indentation depths superpose and follow exactly the same loading curve. The residual depth after complete unloading is larger for deeper indentations. The area under the unloading curve is the reversible elastic strain energy. The area enclosed by the loading and unloading curve is the irreversible energy lost to plastic deformation. The area under the loading curve is the total energy of the indentation. The total energy and the reversible energy are proportional to the cube of the maximum depth 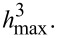


**Figure 3:**
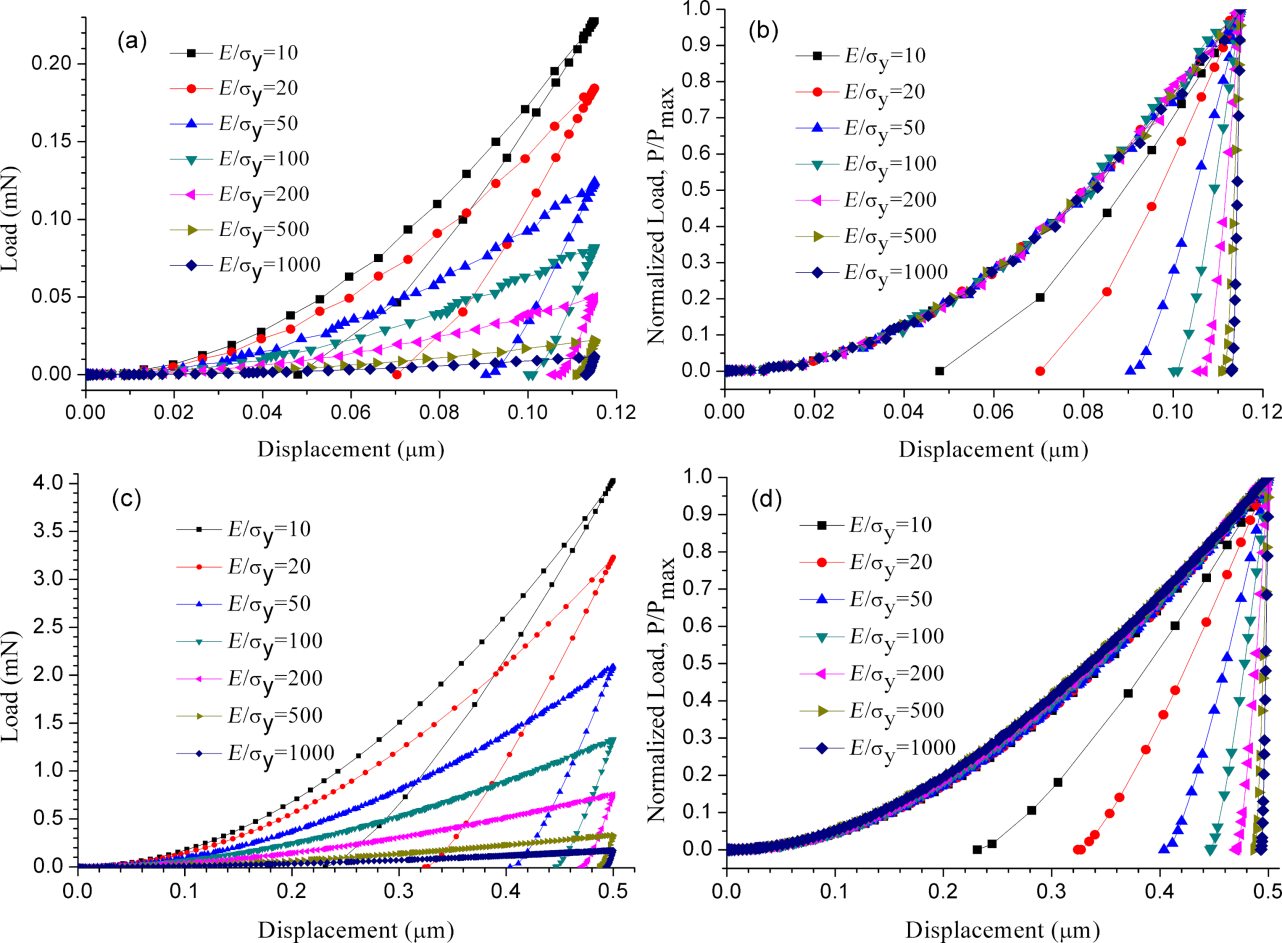
Loading and unloading curves for different *E*/σ_y_: (a) original curves at indentation depth 0.115 µm; (b) curves normalized by the maximum load at indentation depth 0.115 µm; (c) original curves at indentation depth 0.5 µm; (d) curves normalized by the maximum load at indentation depth 0.5 µm.

Figure 3a and Figure 3c show the loading and unloading curves for different *E*/σ_y_ at indentation depths of 0.115 µm and 0.5 µm, respectively. As shown in Figure 3a and Figure 3c, the indenter displacement for *E*/σ_y_ = 200–1000 is plastic, and only a small portion of elasticity is recovered on unloading due to the fact that the deformation of materials with large *E*/σ_y_ is dominated by plasticity. The surface around the indenter piles up. However, the indenter displacement for *E*/σ_y_ = 10–50 is more elastic. Hence, a larger portion of elasticity is recovered on unloading. The surface around the indenter sinks in. For highly elastic solids, such as polymers, sink-in is often observed [5]. *E*/σ_y_ = 100 is a critical value for surface pileup or sink-in. The surface near a conical indenter with a half-apical angle of 70.3° has a tendency to pile up around the indenter and forms a crater when *E*/σ_y_ is greater than 100. However, when *E*/σ_y_ is less than 100, the surface near a conical indenter with a half-apical angle of 70.3° sinks in. The load of each curve in Figure 3b and Figure 3d is normalized with respect to the maximum load from Figure 3a and Figure 3c, respectively. As shown in Figure 3b and Figure 3d, all loading curves superpose. This means that all loading curves in Figure 3a and Figure 3c are proportional for different *E*/σ_y_, and all loading curves for different materials can be generated from a single indentation.

**Figure 4:**
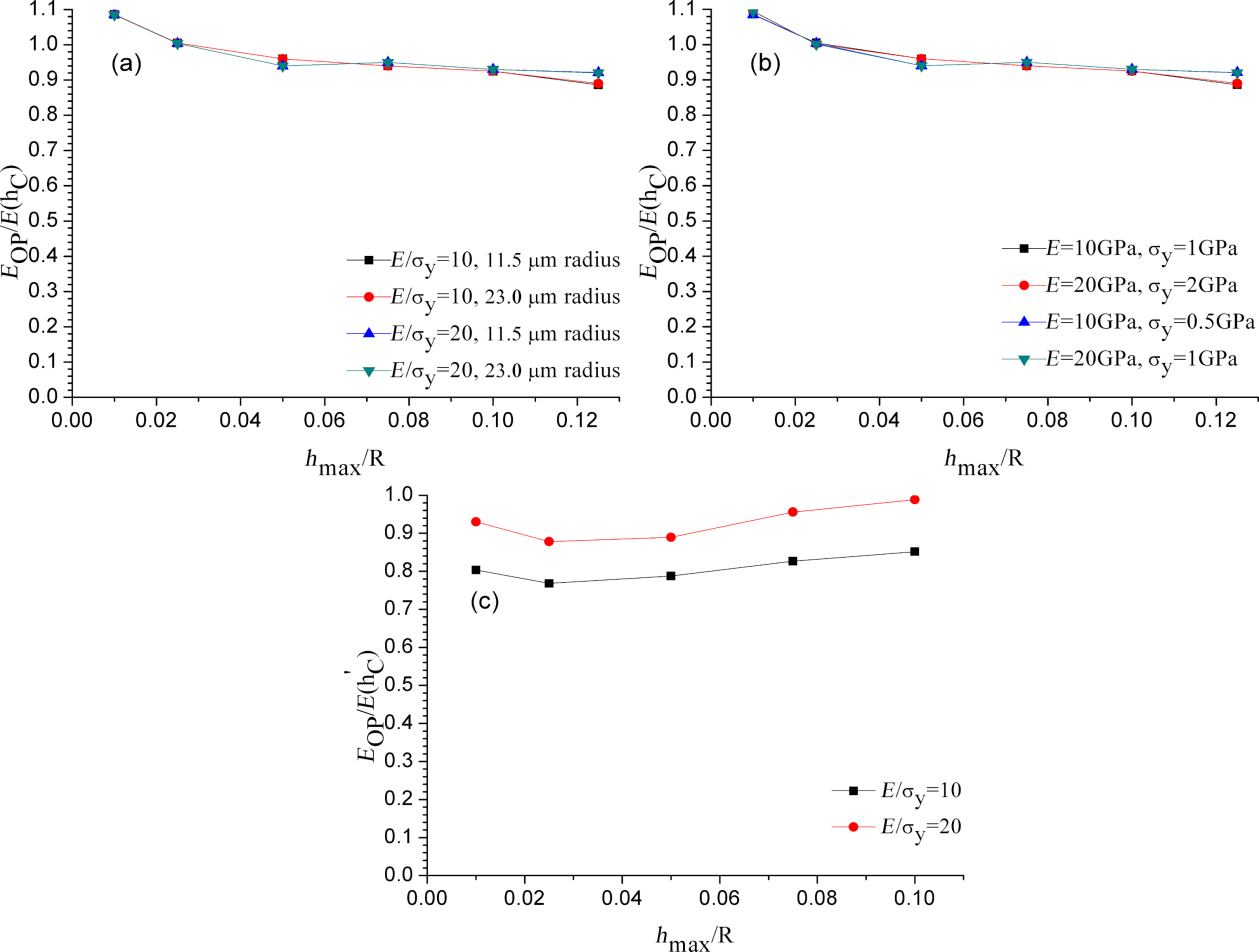
Normalized elastic modulus *E*_OP_/*E* as a function of the maximum indentation depth for different microsphere radii: (a) variable σ_y_, contact depth *h*_C_ is used; (b) variable *E* and σ_y_, contact depth *h*_C_ is used; (c) using the contact height 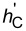 from Equation 11.

Since the elastic modulus is deduced directly from the contact area through Equation 8, the error of the contact area derived from the indentation load–displacement data has important effects for experimentally accurate determinations of the elastic modulus. To show how large the error is, the actual contact areas have been used to calculate the Oliver–Pharr modulus via finite element analyses. Figure 4a shows the elastic modulus extracted using the Oliver–Pharr method normalized with respect to input elastic modulus in the finite element code, *E*_OP_/*E*, as a function of the normalized maximum indentation depth, *h*_max_/*R*. The initial unloading slope was computed using the two points associated with the maximum load and the first unloading point as shown in Figure 2, Figure 3a, and Figure 3c. Then, the Oliver–Pharr modulus *E*_OP_ can be obtained according to Equation 8. The value of contact depth *h*_C_ extracted from finite element simulations is equal to the theoretical value obtained from the Equation 9 as follows:

[10] $$E_{\text{OP}}/E=\beta =\sqrt{\frac{\pi }{24.5}}\frac{\left(1-\nu^{2}\right)}{2E}\frac{S}{h_{\text{C}}},$$

[9] $$h_{\text{C}}=h_{\max}-\frac{2(\pi -2)}{\pi }\frac{P_{\max}}{S}.$$

The values of *E*_OP_/*E* in Figure 4a correspond to the “correction factor” in Equation 10 extracted from the Oliver–Pharr method. The correction factor is not a constant. It decreases with the increase of normalized maximum indentation depth. It demonstrates that the nanoindentation of microspherical materials exhibits a size effect. The deviation in the correction factor as shown in Figure 4a is around 9%. This means that the extracted elastic modulus of a microsphere using the Oliver–Pharr method from the simulated unloading curve depends on the indentation depth. As shown in Figure 4a, the extracted correction factor of nanoindentation simulations on a microsphere of 23 µm radius is completely congruent with that of 11.5 µm radius when the value of maximum indentation depth normalized with respect to microsphere radius is the same. Figure 4b shows that as long as the ratio between elastic modulus of the microsphere and the initial yield stress of the microsphere, *E*/σ_y_, is the same value, the calculated *E*_OP_/*E* is equal for the same indentation depth. This means that as long as *E*/σ_y_ has the same value, it has the same result when *E* or σ_y_ is varied. Taking an example, if σ_y_ = 500 MPa, the elastic modulus is varied from 5 GPa to 500 GPa.

As shown in Figure 4c, the contact depth *h*_C_ is replaced with the contact height 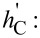


[11] $$h_{\text{C}}^{'}=\cos^{2}\phi \left[\begin{array}{l} h_{\text{max}}-R\\ +{\left(R^{2}+2Rh_{\max}\tan^{2}\phi -h_{\max}^{2}\tan^{2}\phi \right)}^{1/2} \end{array}\right],$$

where ϕ is the half-apical angle of the indenter. A full derivation of the contact height and Equation 11 is available in [26]. As shown in Figure 4c, the deviation of the correction factor to unity is about 23% and 12% for *E*/σ_y_ = 10 and *E*/σ_y_ = 20, respectively. The results show that when the actual contact depth is replaced with the contact height, the material properties of microsphere depend on *E*/σ_y_. Therefore, the contact height is unsuitable to be used to replace the contact depth.

**Figure 5:**
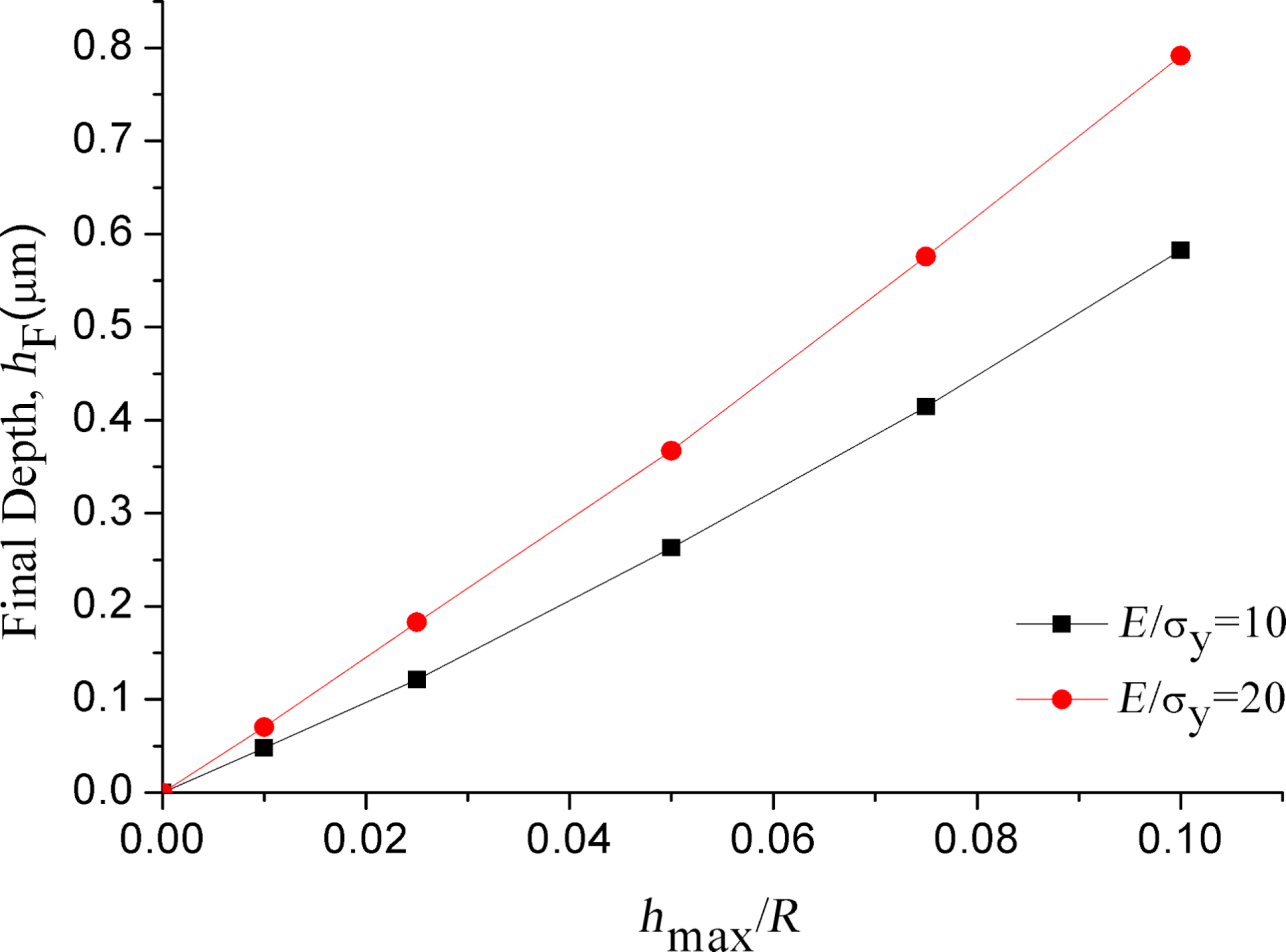
Final depth as a function of maximum indentation depth over microsphere radius.

**Figure 6:**
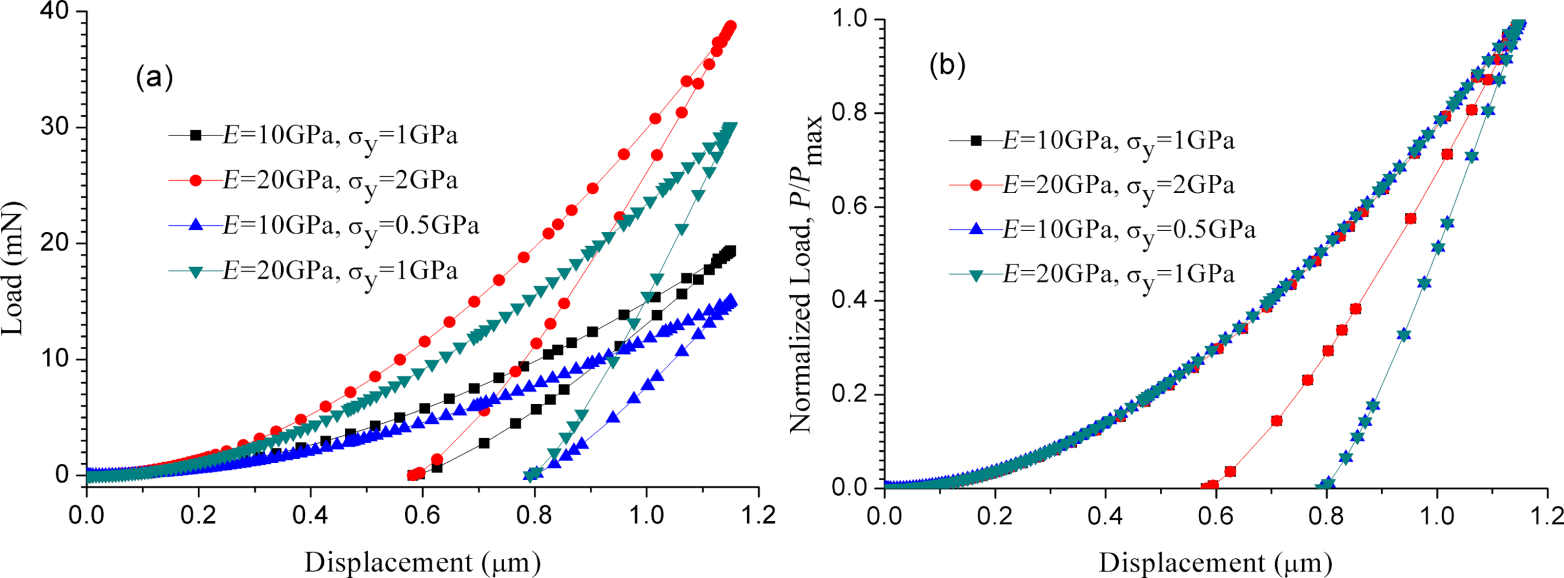
Loading and unloading curves for *E*/σ_y_ = 10 and *E*/σ_y_ = 20 at an indentation depth of 1.15 µm: (a) original curves; (b) curves normalized by the maximum load.

Figure 5 shows the final depth *h*_F_ (the residual depth relative to the initial undeformed surface) as a function of the ratio between the maximum indentation depth and the microsphere radius, *h*_max_/*R*. The final depth increases nonlinear with the increase of *E*/σ_y_ and *h*_max_/*R*. When the value of *E*/σ_y_ is increased from 10 to 20, the final depth increases from 0.58 to 0.79 µm at *h*_max_/*R* = 0.1. Figure 6 shows the loading and unloading curves for *E*/σ_y_ = 10 (*E* = 10 GPa, σ_y_ = 1 GPa; *E* = 20 GPa, σ_y_ = 2 GPa) and *E*/σ_y_ = 20 (*E* = 10 GPa, σ_y_ = 0.5 GPa; *E* =20 GPa, σ_y_ = 1 GPa) at an indentation depth of 1.15 µm. Figure 6a clearly shows that the final depths after indentation are equal for the same values of *E*/σ_y_. As shown in Figure 6b, all loading curves superpose. This substantiates that the loading curve for different materials can be generated from a single indentation.

**Figure 7:**
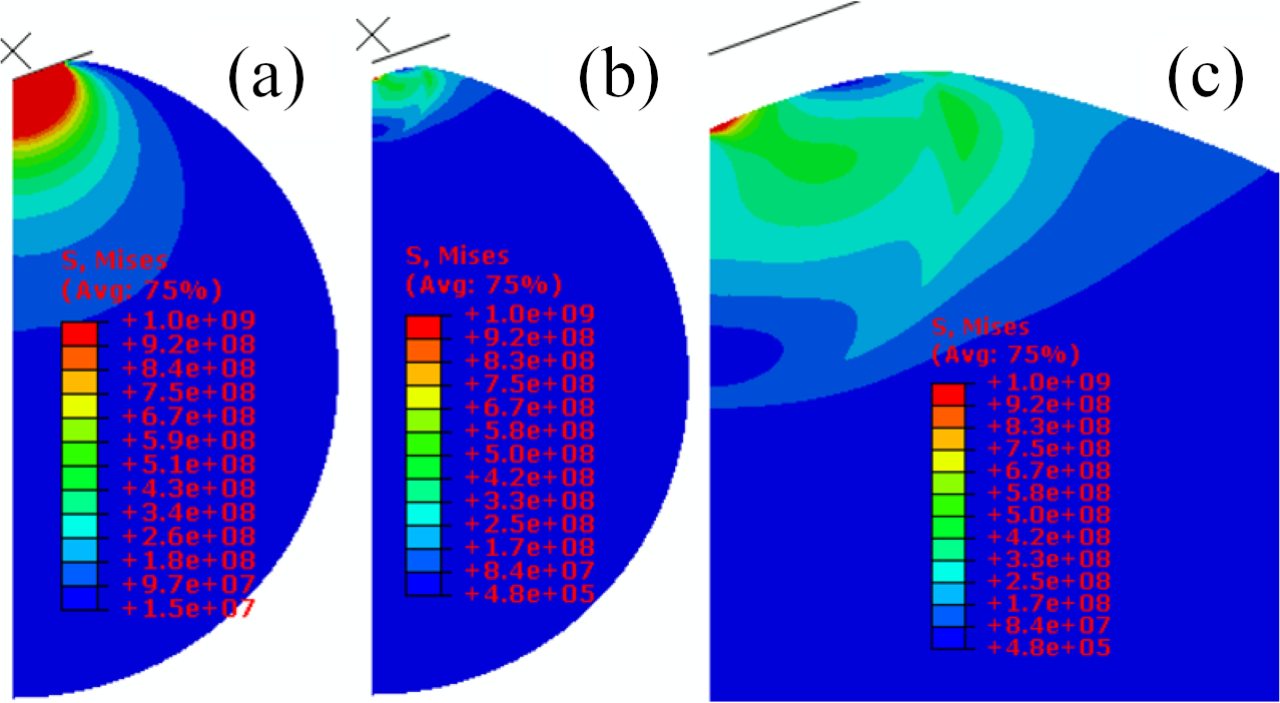
Stress distribution inside a microsphere of 11.5 µm radius for an indentation depth of 1.15 µm at *E*/σ_y_ = 10: (a) at maximum load; (b) after complete unloading; (c) enlarged image of the residual stress distribution.

The stress distribution inside the microsphere at any time during indentation, the residual stress distribution inside the microsphere, and the permanent deformation of the microsphere have been predicted via finite element analyses. Figure 7 shows the stress fields at maximum indentation force, the permanent deformation, and residual stress distributions inside a microsphere of 11.5 µm radius after full unloading. In the purely elastic contact solution, material always sinks in, while for elastic–plastic contact, material may either sink in or pile up. The fundamental material property affecting pileup is the ratio between elastic modulus and yield stress, *E*/σ_y_. Pileup is greater in materials with larger *E*/σ_y_ ratios, such as soft materials. Hard materials and most polymers, ceramics, and glasses have small *E*/σ_y_ ratios. As *E*/σ_y_ decreases, corresponding to increases in the yield stress and decreases in *h*_f_/*h*_max_, the size of the plastic zone decreases until, at some point, the plastic zone boundary at the surface coincides with the contact perimeter indicating the transition from pileup to sink-in behavior. Whether a microspherical material piles up or sinks in during nanoindentation correlates to the size of the plastic zone as shown in Figure 7. As shown in Figure 7c, the surface around the indenter sinks in.

## Conclusion

A systematically computational study has been undertaken to simulate the instrumented nanoindentation of elastic–plastic microspherical materials. The ratio between elastic modulus of the microspherical material and the initial yield stress of the microspherical material was systematically varied from 10 to 1000 to cover the mechanical properties of most materials encountered in engineering. Simulation results indicate that the loading curve for different materials can be generated from a single indentation. The value of contact depth extracted from simulations is equal to the theoretical value. Contact height is unsuitable to be used to replace contact depth for obtaining the indentation elastic modulus of microspherical materials. The calculated elastic modulus of a microspherical material using the Oliver–Pharr method with the simulated unloading curve is found to depend on the indentation depth. This demonstrates that nanoindentation of microspherical materials exhibits a “size effect”.

## Availability of Data and Materials

The raw/processed data that support the findings of this study are available from the corresponding author upon reasonable request although the data also forms part of an ongoing study.

## References

[1] Li H Z, Liu C T, Chen J L (2021). Fibers Polym.

[2] Li H Z, Chen J L, Chen Q H, Liu M (2021). Mater Des.

[3] Oliver W C, Pharr G M (1992). J Mater Res.

[4] Pharr G M, Oliver W C, Brotzen F R (1992). J Mater Res.

[5] Cheng Y-T, Cheng C-M (2004). Mater Sci Eng, R.

[6] Oliver W C, Pharr G M (2004). J Mater Res.

[7] Giannakopoulos A E, Suresh S (1999). Scr Mater.

[8] Dao M, Chollacoop N, Van Vliet K J, Venkatesh T A, Suresh S (2001). Acta Mater.

[9] McElhaney K W, Vlassak J J, Nix W D (1998). J Mater Res.

[10] Bolshakov A, Pharr G M (1998). J Mater Res.

[11] Nix W D, Gao H (1998). J Mech Phys Solids.

[12] Swadener J G, George E P, Pharr G M (2002). J Mech Phys Solids.

[13] Duan S H, Liu F, Pettersson T, Creighton C, Asp L E (2020). Carbon.

[14] Li H Z, Jia Y X, Mamtimin G, Jiang W, An L J (2006). Mater Sci Eng, A.

[15] Yan J, Chen X, Karlsson A M (2007). J Eng Mater Technol.

[16] Li H Z, Li S G, Wang Y C (2011). J Mater Res.

[17] Li H Z, Kandare E, Li S G, Wang Y C, Kandola B K, Myler P, Horrocks A R (2012). Comput Mater Sci.

[18] Phadikar J K, Bogetti T A, Karlsson A M (2012). Int J Solids Struct.

[19] Sudharshan Phani P, Oliver W C (2019). Mater Des.

[20] Breumier S, Villani A, Maurice C, Lévesque M, Kermouche G (2019). Mater Des.

[21] Ecker W, Keckes J, Krobath M, Zalesak J, Daniel R, Rosenthal M, Todt J (2020). Mater Des.

[22] Zhou Y, Fan Q B, Liu X, Wang D D, Zhu X J (2020). Mater Des.

[23] Sanchez-Camargo C-M, Hor A, Salem M, Mabru C (2020). Mater Des.

[24] Goto K, Watanabe I, Ohmura T (2020). Mater Des.

[25] Jeong K, Lee H, Kwon O M, Jung J, Kwon D, Han H N (2020). Mater Des.

[26] McAllister Q P, Gillespie J W, VanLandingham M R (2012). J Mater Res.

[27] Sneddon I N (1965). Int J Eng Sci.

[28] Hay J C, Bolshakov A, Pharr G M (1999). J Mater Res.

